# High quality draft genome sequence of the type strain of *Pseudomonas lutea* OK2^T^, a phosphate-solubilizing rhizospheric bacterium

**DOI:** 10.1186/s40793-016-0173-7

**Published:** 2016-08-23

**Authors:** Yunyoung Kwak, Gun-Seok Park, Jae-Ho Shin

**Affiliations:** School of Applied Biosciences, College of Agriculture and Life Sciences, Kyungpook National University, Daegu, 702-701 Republic of Korea

**Keywords:** *Pseudomonad*, Phosphate-solubilizing, Plant growth promoting rhizobacteria (PGPR), Biofertilizer

## Abstract

*Pseudomonas lutea* OK2^T^ (=LMG 21974^T^, CECT 5822^T^) is the type strain of the species and was isolated from the rhizosphere of grass growing in Spain in 2003 based on its phosphate-solubilizing capacity. In order to identify the functional significance of phosphate solubilization in *Pseudomonas* Plant growth promoting rhizobacteria, we describe here the phenotypic characteristics of strain OK2^T^ along with its high-quality draft genome sequence, its annotation, and analysis. The genome is comprised of 5,647,497 bp with 60.15 % G + C content. The sequence includes 4,846 protein-coding genes and 95 RNA genes.

## Introduction

Phosphorus, one of the major essential macronutrients for plant growth and development, is usually found in insufficient quantities in soil because of its low solubility and fixation [1, 2]. Since phosphorus deficiency in agricultural soil is limits plant growth, the release bound phosphorus from soils by microbes is an important aspect that can be used to improve soil fertility for increasing crop yields [2].

Phosphate-solubilizing microorganisms, a group of soil microorganisms capable of converting insoluble phosphate to soluble forms, have received attention as efficient bio-fertilizers for enhancing the phosphate availability for plants [3]. As one of the representative phosphate-solubilizing bacteria [4], rhizosphere-colonizing pseudomonads are of interest owing to the benefits they offer to plants. Besides increasing the phosphate accessibility, they promote plant development by facilitating direct and indirect plant growth promotion through the production of phytohormones and enzymes or through the suppression of soil-borne diseases by inducing systemic resistance in the plants [5–7].

*Pseudomonas lutea* OK2^T^ (=LMG 21974^T^, CECT 5822^T^) with insoluble phosphate-solubilizing activity was isolated from the rhizosphere of grass growing in northern Spain [8]. Characteristics of the whole genome sequence and a brief summary of the phenotype for this type strain are presented in this study.

## Organism information

### Classification and features

**Fig. 1:**
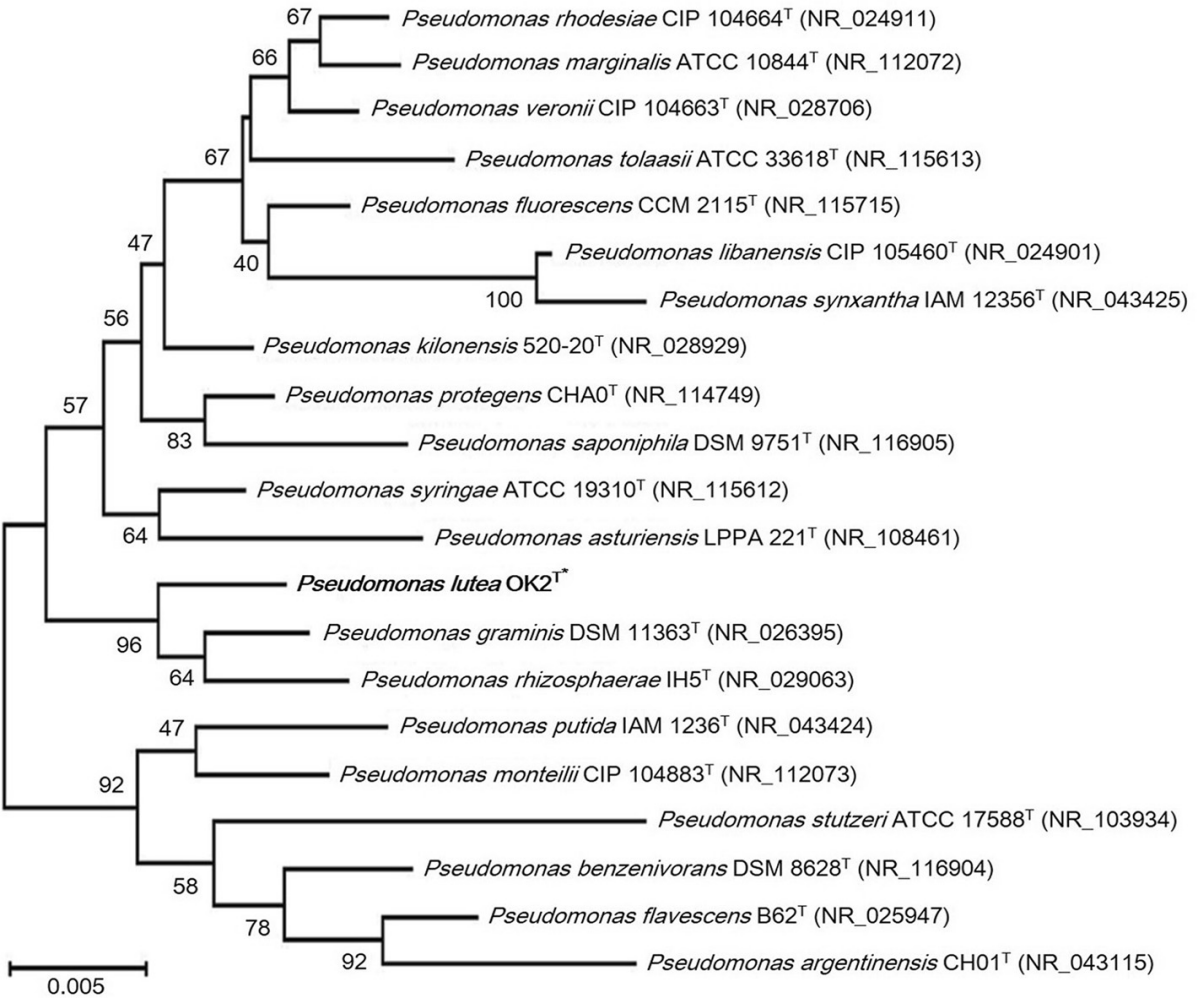
A phylogenetic tree constructed using the neighbor-joining method presenting the position of *Pseudomonas lutea* OK2^T^ (shown in bold print with asterisk) relative to the other species within the genus *Pseudomonas*. Only the type strains from the genus *Pseudomonas* presenting full-length 16S rRNA gene sequences were selected from the NCBI database [43]. The nucleotide sequences of these strains were aligned using CLUSTALW [44], and a phylogenetic tree was constructed with the MEGA version 6 package [45] using the neighbor-joining method with 1,000 bootstrap replicates [16]. The bootstrap values for each species are indicated at the nodes. Scale bar indicates 0.005 nucleotide change per nucleotide position. The strains selected for the analysis of the 16S rRNA gene and their corresponding GenBank accession numbers are as follows: *Pseudomonas rhodesiae* CIP 104664^T^ (NR_024911) [14, 46]; *Pseudomonas marginalis* ATCC 10844^T^ (NR_112072) [47, 48]; *Pseudomonas veronii* CIP 104663^T^ (NR_028706) [49]; *Pseudomonas tolaasii* ATCC 33618^T^ (NR_115613) [47, 50]; *Pseudomonas fluorescens* CCM 2115^T^ (NR_115715) [47, 51]; *Pseudomonas libanensis* CIP 105460^T^ (NR_024901) [52]; *Pseudomonas synxantha* IAM 12356^T^ (NR_043425) [47, 53]; *Pseudomonas kilonensis* 520-20^T^ (NR_028929) [54]; *Pseudomonas protegens* CHA0^T^ (NR_114749) [13, 55]; *Pseudomonas saponiphila* DSM 9751^T^ (NR_116905) [56, 57]; *Pseudomonas syringae* ATCC 19310^T^ (NR_115612) [47, 58]; *Pseudomonas asturiensis* LPPA 221^T^ (NR_108461) [59]; *Pseudomonas graminis* DSM 11363^T^ (NR_026395) [10]; *Pseudomonas rhizosphaerae* IH5^T^ (NR_029063) [11]; *Pseudomonas putida* IAM 1236^T^ (NR_043424) [47, 60]; *Pseudomonas monteilii* CIP 104883^T^ (NR_112073) [61]; *Pseudomonas stutzeri* ATCC 17588^T^ (NR_103934) [47, 62]; *Pseudomonas benzenivorans* DSM 8628^T^ (NR_116904) [56, 57]; *Pseudomonas flavescens* B62^T^ (NR_025947) [63]; and *Pseudomonas argentinensis* CH01^T^ (NR_043115) [15]

A 16S rRNA gene sequence of *P. lutea* OK2^T^ was compared to those of other type strains of the genus *Pseudomonas* using BLAST on NCBI [9]. The 16S rRNA gene sequence showed highest similarity (99 % identity) to that of *P. graminis*DSM 11363^T^ [10], followed by similarity to the 16S rRNA gene sequence of *P. rhizosphaerae*IH5^T^ (98 % identity) [11], *P. protegens*CHA0^T^ (98 % identity) [12, 13], *P. rhodesiae*CIP 104664^T^ (97 % identity) [14], and *P. argentinensis*CH01^T^ (97 % identity) [15]. Species showing full-length 16S rRNA gene sequences in BLAST analysis were considered for further phylogenetic analyses. A phylogenetic tree was constructed using the neighbor-joining method [16], and the bootstrap value was set as 1,000 times random replicate sampling. The consensus phylogenetic neighborhood of *P. lutea* OK2^T^ within the genus *Pseudomonas* is shown in Fig. 1.

**Fig. 2:**
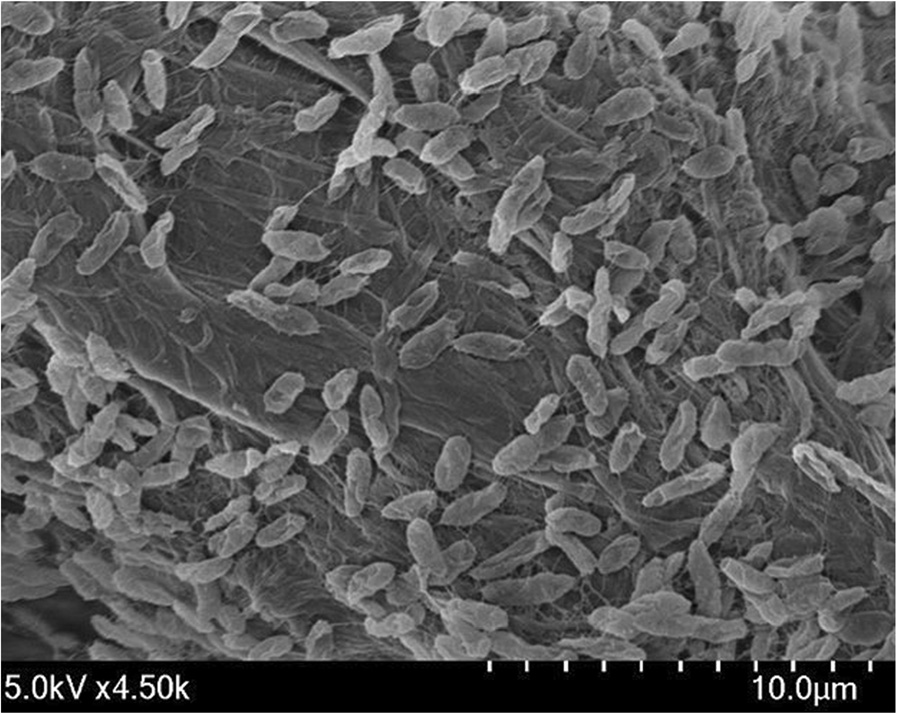
Scanning electron micrograph of *Pseudomonas lutea* OK2^T^. The image was taken under a Field Emission Scanning Electron Microscope (FE-SEM, SU8220; Hitachi, Japan) at an operating voltage of 5.0 kV. The scale bar represents 10.0 μm

*P. lutea* OK2^T^ is a motile, strictly aerobic, non-spore forming, gram-negative bacterium that belongs to the family *Pseudomonadaceae* of the class *Gammaproteobacteria* [8]. The cells are rod-shaped with a diameter of approximately 0.75 μm and a length of 1.2–1.6 μm (Fig. 2). The strain produces yellow, translucent, circular convex colonies of 1–2 mm diameter on plates containing YED-P medium (per liter: 7.0 g of glucose, 3.0 g of yeast extract, 3.0 g of bicalcium phosphate, and 17.0 g of agar) within 2 days at 25 °C [8]. *P. lutea* OK2^T^ is capable of oxidizing glucose in media containing ammonium nitrate as a nitrogen source and hydrolyzes aesculin [8]. The strain OK2^T^ is positive for catalase, but negative for oxidase, gelatinase, caseinase, urease, β-galactosidase, arginine dehydrolase, tryptophan deaminase, and indole/H_2_S [8]. Further, it can utilize galactose, ribose, mannose, glycerol, D-fructose, D-xylose, D-/L-arabinose, D-/L-arabitol, D-/L-fucose, L-lyxose, melibiose, inositol, mannitol, adonitol, xylitol, caprate, malate, gluconate, 2-ketogluconate, and citrate as sole carbon sources, but cannot utilize maltose, lactose, sucrose, trehalose, cellobiose, starch, glycogen, inulin, sorbitol, D-tagatose, D-raffinose, L-xylose, L-sorbose, L-rhamnose, N-acetylglucosamine, salicin, and erythritol [8]. Unlike other pseuodomonads, the strain OK2^T^ does not produce fluorescent pigments [8].

#### Chemotaxonomic data

The important non-polar fatty acids present in *P. lutea* OK2^T^ include hexadecenoic acid (16:1, 39.0 %), hexadecanoic acid (16:0, 29.0 %), and octadecenoic acid (18:1, 18.6 %). In addition, the strain OK2^T^ has hydroxy fatty acids such as 3-hydroxydodecanoic acid (3-OH 12:0, 3.3 %), 2-hydroxydodecanoic acid (2-OH 12:0, 2.7 %), and 3-hydroxydecanoic acid (3-OH 10:0, 2.4 %) [8]. The whole-cell fatty acid profile of this strain is similar to that observed in other representative strains of the genus *Pseudomonas*, such as *P. graminis* [10] and *P. rhizosphaerae* [11]. The general characteristics of the strain are summarized in Table 1.

**Table 1:** Classification and general features of *Pseudomonas lutea* OK2^T^ [18]

MIGS ID	Property	Term	Evidence code^a^
	Classification	Domain *Bacteria*	TAS [64]
		Phylum *Proteobacteria*	TAS [65]
		Class *Gammaproteobacteria*	TAS [66, 67]
		Order *Pseudomonadales*	TAS [47, 68, 69]
		Family *Pseudomonadaceae*	TAS [47, 70]
		Genus *Pseudomonas*	TAS [47, 71–73]
		Species *Pseudomonas lutea*	TAS [8]
		Type strain OK2^T^ (=LMG 21974^T^, CECT 5822^T^)	TAS [8]
	Gram stain	Negative	TAS [8, 74]
	Cell shape	Rod-shaped	TAS [8, 74]
	Motility	Motile	TAS [8, 74]
	Sporulation	None	TAS [8, 74]
	Temperature range	Mesophilic	NAS
	Optimum temperature	25°C	TAS [8]
	pH range	7.0–7.5	NAS
	Carbon source	Heterotrophic	TAS [75]
MIGS-6	Habitat	Soil	TAS [8]
MIGS-6.3	Salinity	Not reported	
MIGS-22	Oxygen requirement	Aerobic	TAS [8, 74]
MIGS-15	Biotic relationships	Free living	NAS
MIGS-14	Pathogenicity	Non-pathogen	
MIGS-4	Geographic location	Spain; northern Spain	TAS [8]
MIGS-5	Sample collection	2003	NAS
MIGS-4.1	Latitude	Not reported	
MIGS-4.2	Longitude	Not reported	
MIGS-4.4	Altitude	Not reported	

^a^Evidence codes - IDA: Inferred from Direct Assay; TAS: Traceable Author Statement (i.e., a direct report exists in the literature); NAS: Non-traceable Author Statement (i.e., not directly observed for the living, isolated sample, but based on a generally accepted property for the species, or anecdotal evidence). These evidence codes are from the Gene Ontology project [76]

## Genome sequencing information

### Genome project history

*P. lutea* OK2^T^ was selected as a novel-phosphate solubilizing strain for the genome-sequencing project of agriculturally useful microbes undertaken at Kyungpook National University. Genome sequencing was performed in September 2014, and the results of the Whole Genome Shotgun project have been deposited at DDBJ/EMBL/GenBank under the accession number JRMB00000000. The version described in this study is the first version, indicated as JRMB00000000.1. The information obtained from the genome sequencing project is registered on the Genome Online Database [17] with the GOLD Project ID Gp0107463. A summary of this information and its association with the Minimum Information about a Genome Sequence (MIGS) version 2.0 compliance [18] are presented in Table 2.

**Table 2:** Project information

MIGS ID	Property	Term
MIGS-31	Finishing quality	Draft
MIGS-28	Libraries used	10-kb SMRT-bell library
MIGS-29	Sequencing platforms	PacBio RS II
MIGS-31.2	Fold coverage	67.58 ×
MIGS-30	Assemblers	RS HGAP Assembly Protocol [20] in SMRT analysis pipeline v.2.2.0
MIGS-32	Gene calling method	NCBI Prokaryotic Genome Annotation Pipeline [77]; GeneMarkS+ [78]
	Locus Tag	LT42
	Genbank ID	JRMB00000000
	Genbank Date of Release	September 29, 2014
	GOLD ID	Gp0107463
	BIOPROJECT	PRJNA261881
MIGS-13	Source material identifier	LMG 21974^T^, CECT 5822^T^
	Project relevance	Agriculture

### Growth conditions and genomic DNA preparation

The strain was cultured in tryptic soy broth (Difco Laboratories Inc., Detroit, MI) at 30 °C on a rotary shaker at 200 rpm. Genomic DNA was isolated using a QIAamp® DNA Mini Kit (Qiagen, Valencia, CA) according to the manufacturer's standard protocol. The quantity and purity of the extracted genomic DNA were assessed using a Picodrop Microliter UV/Vis Spectrophotometer (Thermo Fisher Scientific Inc., Waltham, MA) and Qubit® 2.0 Fluorometer (Fisher Scientific Inc., Pittsburgh, PA), respectively.

### Genome sequencing and assembly

The isolated genomic DNA of *P. lutea* OK2^T^ was sequenced using the SMRT DNA sequencing platform and the Pacific Biosciences RS II sequencer with P4 polymerase-C2 sequencing chemistry (Pacific Biosciences, Menlo Park, CA) [19]. After shearing the genomic DNA, a 10-kb insert SMRT-bell library was prepared and loaded on two SMRT cells. During the 90 min of movie time, 654,270,150 read bases were generated from 300,584 reads. All the obtained bases were filtered to remove any reads shorter than 100 bp or those having accuracy values less than 0.8. Subsequently, 461,880,761 nucleotides were obtained from 116,562 reads, with a read quality of 0.843. These bases were assembled *de novo* using the RS HGAP assembly protocol version 3.3 on the SMRT analysis platform version 2.2.0 [20]. The HGAP analysis yielded five contigs corresponding to five scaffolds, with a 67.58-fold coverage. The maximum contig length and N50 contig length were identical: 2,839,280 bp. The total length of the *P. lutea* OK2^T^ genome was found to be 5,647,497 bp.

### Genome annotation

The protein coding sequences were determined using the NCBI PGAP version 2.8 (rev. 447021) [21]. Additional gene prediction and functional annotation analyses were performed on the RAST server [22] and IMG-ER pipeline, respectively, by the Department of Energy-Joint Genome Institute [23].

## Genome properties

**Fig. 3:**
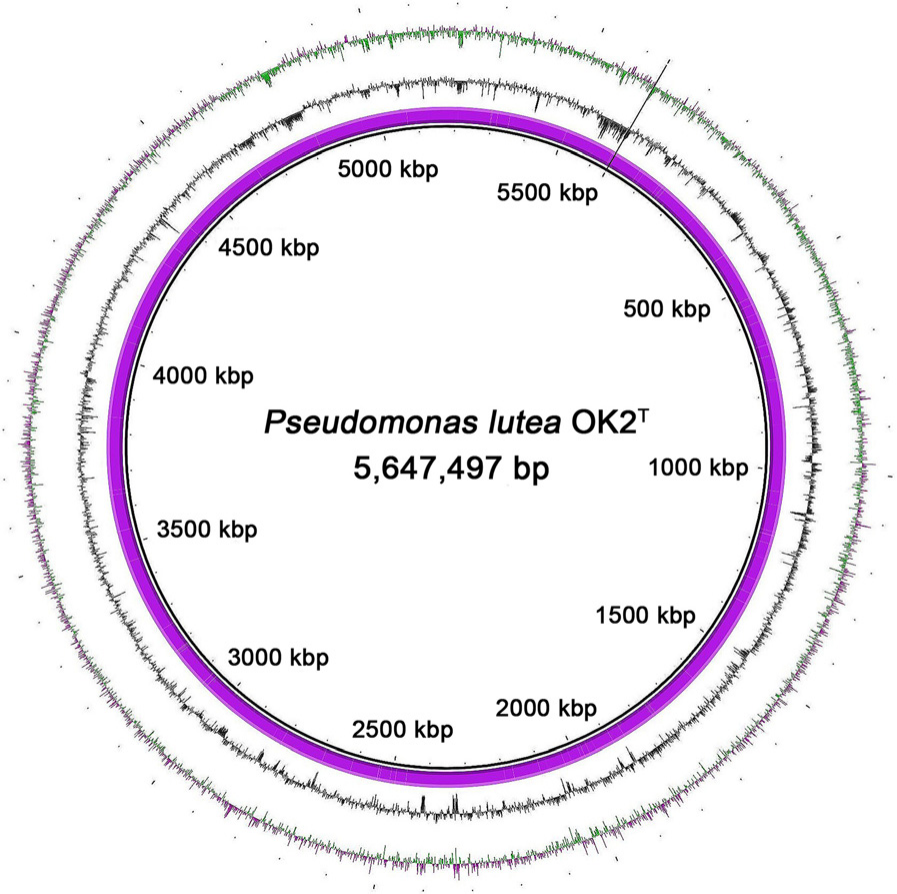
Graphical circular map of the *Pseudomonas lutea* OK2^T^ genome. The circular map was generated using the BLAST Ring Image Generator program [79]. From the inner circle to the outer circle: Genetic regions; GC content (*black*); and GC skew (*purple*/*green*)

The average G + C content of the genome was 60.15 %. The genome was predicted to encode 4,941 genes including 4,846 protein-coding genes and 95 RNA genes (24 rRNAs, 70 tRNAs, and 1 ncRNA). Putative functions were assigned to 4,102 of the protein-coding genes, and 3,507 genes (approximately 70.98 %) were assigned to the COG functional categories. The most abundant COG category was "Amino acid transport and metabolism" (10.36 %), followed by "General function prediction only" (8.71 %), “Transcription" (8.34 %), and “Signal transduction mechanisms” (6.52 %). The category for “Mobilome: prophages, transposons” (0.92 %) was also classified with functional genes for transposase (LT42_00515, LT42_05870, LT42_07855, LT42_10965, LT42_14240, LT42_14330, LT42_18595, LT42_19270, LT42_21870, LT42_21925), integrase (LT42_17205), terminase (LT42_06460, LT42_17145, LT42_17150), and plasmid stabilization protein (LT42_19025, LT42_24175). The genome statistics of strain OK2^T^ are presented in Table 3 and Fig. 3. The gene distribution within the COG functional categories is presented in Table 4.

**Table 3:** Genome statistics

Attribute	Value	% of Total
Genome size (bp)	5,647,497	100.00
DNA coding (bp)	4,778,153	84.61
DNA G + C (bp)	3,397,087	60.15
DNA scaffolds	5	100.00
Total genes	4,941	100.00
Protein coding genes	4,846	98.08
RNA genes	95	1.92
Pseudo genes	239	4.84
Genes in internal clusters	1,402	26.64
Genes with function prediction	4,102	83.02
Genes assigned to COGs	3,507	70.98
Genes with Pfam domains	4,026	81.48
Genes with signal peptides	485	9.82
Genes with transmembrane helices	1,026	20.77
CRISPR repeats	0	0.00

**Table 4:** Number of genes associated with general COG functional categories

Code	Value	% age	Description
J	231	5.75	Translation, ribosomal structure and biogenesis
A	1	0.02	RNA processing and modification
K	335	8.34	Transcription
L	121	3.01	Replication, recombination and repair
B	2	0.05	Chromatin structure and dynamics
D	34	0.85	Cell cycle control, Cell division, chromosome partitioning
V	73	1.82	Defense mechanisms
T	262	6.52	Signal transduction mechanisms
M	228	5.68	Cell wall/membrane biogenesis
N	133	3.31	Cell motility
U	97	2.41	Intracellular trafficking and secretion
O	152	3.78	Posttranslational modification, protein turnover, chaperones
C	248	6.17	Energy production and conversion
G	256	6.37	Carbohydrate transport and metabolism
E	416	10.36	Amino acid transport and metabolism
F	85	2.12	Nucleotide transport and metabolism
H	198	4.93	Coenzyme transport and metabolism
I	182	4.53	Lipid transport and metabolism
P	234	5.83	Inorganic ion transport and metabolism
Q	98	2.44	Secondary metabolites biosynthesis, transport and catabolism
R	350	8.71	General function prediction only
S	212	5.28	Function unknown
-	1434	29.02	Not in COGs

The total is based on the total number of protein coding genes in the genome

## Insights from the genome sequence

Microorganisms that show phosphate-solubilizing activity are generally known to be involved in either of the following two biochemical mechanisms: production of organic acids for the acidification of external surroundings for plants and production of enzymes for direct solubilization [24, 25]. Genes encoding functional enzymes with these biochemical properties were predicted using the KO database via IMG-ER pipeline [26, 27]. The genome of *P. lutea* OK2^T^ was annotated with several genes involved in phosphate solubilization. For example, *ldhA* (D-lactate dehydrogenase, KO:K03778) and *icd* (isocitrate dehydrogenase, KO:K00031) were found to be involved in the production of organic acids, and *phoD* (alkaline phosphatase D, KO:K01113) was involved in direct phosphate solubilization. Direct oxidation of glucose to gluconic acid by a periplasmic membrane-bound glucose dehydrogenase is also known to be one of the major metabolic steps for phosphate solubilization in pseudomonads [6]. In relation to this process, the *gcd* gene coding for a cofactor pyrroloquinoline quinone-dependent glucose dehydrogenase (=quinoprotein glucose dehydrogenase, KO:K00117) was revealed (Table 5). Phosphate solubilization is normally a complex phenomenon depending on conditions such as bacterial, nutritional, physiological, and growth variations [2]. Given that phosphate solubilization can occur through various microbial processes/mechanisms [28], the predicted genes on the genome being described could compositely contribute to this activity.

**Table 5:** Putative genes related to functional enzymes for potential PGPR effects predicted from the genome sequence of *Pseudomonas lutea* OK2^T^

Function ID	Name
Phosphate solubilization	
KO:K01113	alkaline phosphatase D [EC:3.1.3.1] (*phoD*)
KO:K03778*	D-lactate dehydrogenase [EC:1.1.1.28] (*ldhA*) *
KO:K00031	isocitrate dehydrogenase [EC:1.1.1.42] (*icd*)
KO:K01647	citrate synthase [EC:2.3.3.1] (*gltA*)
KO:K00117	quinoprotein glucose dehydrogenase [EC:1.1.5.2] (*gcd*)
Antibiotic resistance	
KO:K17836*	beta-lactamase class A (penicillinase) [EC:3.5.2.6] (*penP*) *
KO:K08218	MFS transporter, PAT family, beta-lactamase induction signal transducer AmpG (*ampG*)
KO:K03806	beta-lactamase expression regulator, N-acetyl-anhydromuramyl-L-alanine amidase AmpD protein (*ampD*)
KO:K03807	Membrane protein required for beta-lactamase induction, AmpE protein (*ampE*)
KO:K05365	penicillin-binding protein 1B [EC:2.4.1.129 3.4.-.-] (*mrcB*)
KO:K05366	penicillin-binding protein 1A [EC:2.4.1.-3.4.-.-] (*mrcA*)
KO:K05367	penicillin-binding protein 1C [EC:2.4.1.-] (*pbpC*)
KO:K05515	penicillin-binding protein 2 (*mrdA*)
KO:K07552	MFS transporter, DHA1 family, bicyclomycin/chloramphenicol resistance protein (*bcr*)
KO:K08223	MFS transporter, FSR family, fosmidomycin resistance protein (*fsr*)
KO:K05595*	multiple antibiotic resistance protein (*marC*) *
KO:K18138	multidrug efflux pump (*acrB*, *mexB*, *adeJ*, *smeE*, *mtrD*, *cmeB*)
KO:K07799	putative multidrug efflux transporter MdtA (*mdtA*)
KO:K07788	RND superfamily, multidrug transport protein MdtB (*mdtB*)
KO:K07789	RND superfamily, multidrug transport protein MdtC (*mdtC*)
Toxins	
KO:K11068	membrane damaging toxins Type II toxin, pore-forming toxin hemolysin III (*hlyIII*)
Metal ion resistance	
KO:K07213	copper chaperone
KO:K07245	putative copper resistance protein D (*pcoD*)
KO:K07665	two-component system, OmpR family, copper resistance phosphate regulon response regulator CusR (*cusR*)
KO:K06189	magnesium and cobalt transporter (*corC*)
KO:K08970*	nickel/cobalt exporter (*rcnA*) *
KO:K06213	magnesium transporter (*mgtE*)
KO:K16074	zinc transporter (*zntB*)
KO:K09815	zinc transport system substrate-binding protein (*znuA*)
KO:K09816	zinc transport system permease protein (*znuB*)
KO:K09823	Fur family transcriptional regulator, zinc uptake regulator (*zur*)
KO:K03893	arsenical pump membrane protein (*arsB*)
KO:K11811*	arsenical resistance protein ArsH (*arsH*) *
Siderophore	
KO:K02362	enterobactin synthetase component D [EC:2.7.8.-] (*entD*)
KO:K16090	catecholate siderophore receptor (*fiu*)
Attachment and colonization in the plant rhizosphere	
KO:K04095*	cell filamentation protein (*fic*) *
KO:K06596*	chemosensory pili system protein ChpA (sensor histidine kinase/response regulator) (*chpA*) *
KO:K02655, K02656, K02662, K02663, K02664, K02665, K02666, K02671, K02672, K02673, K02674, K02676, K02650*, K02652, K02653	type IV pilus assembly protein PilE (*pilE*), PilF (*pilF*), PilM (*pilM*), PilN (*pilN*), PilO (*pilO*), PilP (*pilP*), PilQ (*pilQ*), PilV (*pilV*), PilW (*pilW*), PilX (*pilX*), PilY1 (*pilY1*), PilZ (*pilZ*), PilA (*pilA*)*, PilB (*pilB*), PilC (*pilC*)
KO:K08086, K02280	pilus assembly protein FimV (*fimV*), CpaC (*cpaC*)
KO:K02657, K02658	twitching motility two-component system response regulator PilG (*pilG*), PilH (*pilH*)
KO:K02659, K02660, K02669, K02670*	twitching motility protein PilI (*pilI*), PilJ (*pilJ*), PilT (*pilT*), PilU (*pilU*) *
Secretion system	
KO:K03196*, K03198*, K03199*, K03200*, K03203*, K03204*, K03205*	type IV secretion system protein VirB11 (*virB11*) *, VirB3 (*virB3*) *, VirB4 (*virB4*) *, VirB5 (*virB5*) *, VirB8 (*virB8*) *, VirB9 (*virB9*) *, VirD4 (*virD4*) *
KO:K11891*, K11892*, K11893*, K11894*, K11895*, K11896*, K11900*, K11901*	type VI secretion system protein ImpL (*impL*) *, ImpK (*impK*) *, ImpJ (*impJ*) *, ImpI (*impI*) *, ImpH (*impH*) *, ImpG (*impG*) *, ImpC (*impC*) *, ImpB (*impB*) *
KO:K11903*, K11904*	type VI secretion system secreted protein Hcp (*hcp*) *, VgrG (*vgrG*) *
KO:K11905*	type VI secretion system protein*
KO:K11906*, K11907*, K11910*	type VI secretion system protein VasD (*vasD*) *, VasG (*vasG*) *, VasJ (*vasJ*) *
Plant hormone auxin biosynthesis	
KO:K01696	tryptophan synthase [EC:4.2.1.20] (*trpB*)
KO:K00766	anthranilate phosphoribosyltransferase [EC:2.4.2.18] (*trpD*)
KO:K01817	phosphoribosylanthranilate isomerase [EC:5.3.1.24] (*trpF*)

^a^Based on the function profiles obtained from the KO database [25, 26], under the IMG-ER pipeline [23]

*Predicted only in the genome sequence of *P. lutea* OK2^T^ (IMG Genome ID 2593339262) upon comparison with the complete genome sequence of *P. rhizosphaerae* IH5^T^ (=DSM 16299^T^, IMG Genome ID 2593339263) [34]

*P. lutea* OK2^T^ is also expected to possess functional traits related to plant growth promotion [29–32]. As shown in Table 5, genes coding for functional enzymes with various PGPR effects such as “antibiotic resistance”, “metal ion resistance”, “toxin production”, “siderophore production”, “attachment and colonization in the plant rhizosphere”, and “plant hormone auxin production” were revealed. Although *nif* gene clusters involved in nitrogen-fixing activity were not found in the strain OK2^T^, a gene encoding for the nitrogen-fixation protein NifU (KO:K04488) was identified [33].

Within the genus *Pseudomonas**sensu stricto*, *P. lutea* OK2^T^ is presented as a group phylogenetically closest to *P. graminis*DSM 11363^T^ [10] and *P. rhizosphaerae* IH5^T^ [11] (shown in Fig. 1). The majority of the genes in *P. lutea* OK2^T^ were predicted based on the genome of *P. rhizosphaerae* IH5^T^ (=DSM 16299^T^, IMG Genome ID 2593339263) [34]. However, genes such as *ldhA* (D-lactate dehydrogenase, KO:K03778), *penP* (beta-lactamase class A, KO:K17836), *marC* (multiple antibiotic resistance protein, KO:K05595), *rcnA* (nickel/cobalt exporter, KO:K08970), *arsH* (arsenical resistance protein ArsH, KO:K11811), *fic* (cell filamentation protein, KO:K04095), and *chpA* (chemosensory pili system protein ChpA, KO:K06596) and the gene clusters coding for enzymes with type IV secretion systems were only annotated in OK2^T^. Furthermore, pertinent gene clusters for type VI secretion systems, known as a complex multicomponent secretion machine, with bacterial competitions [35–37] were only predicted in the strain OK2^T^. The type VI secretion system may be related to possible features of bacterial motility/adaptation/competition in the strain. Although the strain *P. graminis*DSM 11363^T^ had similar general features and biochemical properties as strain OK2^T^, its genome sequence is not yet available.

Average Nucleotide Identity calculations [38] were used to compare the genomes of *P. lutea* OK2^T^ and other sequenced *Pseudomonas* species (Table 6). The strain was found to be most closely related to *Pseudomonas syringae*ATCC 19310^T^ (77.31 % identity), followed by *Pseudomonas kilonensis* 520-20^T^ (76.96 % identity). These values are under the acceptable range of species cutoff values of 95–96 % [39], indicating that *P. lutea* OK2^T^ is different from other sequenced *Pseudomonas* species.

**Table 6:** Average nucleotide identity of the genome sequence of different *Pseudomonas* species with that of OK2^T^

Strain	Average Nucleotide Identity (%)
*Pseudomonas syringae* ATCC 19310^T^	77.31
*Pseudomonas kilonensis* 520-20^T^	76.96
*Pseudomonas protegens* CHA0^T^	76.86
*Pseudomonas veronii* CIP 104663^T^	76.72
*Pseudomonas libanensis* CIP 105460^T^	76.48
*Pseudomonas fluorescens* CCM 2115^T^	76.45
*Pseudomonas synxantha* IAM 12356^T^	76.39
*Pseudomonas rhizosphaerae* IH5^T^	76.39
*Pseudomonas putida* IAM 1236^T^	75.59
*Pseudomonas monteilii* CIP 104883^T^	75.39
*Pseudomonas stutzeri* ATCC 17588^T^	73.85

## Conclusions

We presented here the first genome sequence of *P. lutea* OK2^T^, a phosphate-solubilizing bacterium isolated from the rhizosphere of grass in northern Spain [8]. This study showed that *P. lutea* OK2^T^ has potential traits including phosphate-solubilizing capability, making it as an effective pseudomonad-PGPR.

Considering a variety of complex conditions that occur in rhizospheres [40], the environmental adaptability of PGPR in *in situ* rhizosphere became an important factor for improved plant growth-promoting capacity. In addition, initial studies focusing on the functional properties of PGPR [31, 32] have led to interest in the comparative analyses of pan-/core-genomes of these bacteria, which are of ecological importance for elucidating the fundamental genotypic features of PGPR under diverse rhizosphere conditions [41, 42]. The genetic information obtained for *P. lutea* OK2^T^ will improve our understanding of the genetic basis of phosphate-solubilizing pseudomonad-PGPR activities and further provide insights into the practical applications of the strain as a biocontrol agent in the field of agriculture.

## Abbreviations

HGAP: Hierarchical genome assembly process
IMG-ER: Integrated microbial genomes-expert review
KO: Kyoto encyclopedia of genes and genomes Orthology
PGAP: Prokaryotic genome annotation pipeline
PGPR: Plant growth-promoting rhizobacteria
RAST: Rapid annotation using subsystems technology
SMRT: Single molecule real-time
